# Glucocorticoid use and ischemia‐reperfusion injury in laparoscopic liver resection: Randomized controlled trial

**DOI:** 10.1002/ags3.12298

**Published:** 2019-11-19

**Authors:** Yasushi Hasegawa, Hiroyuki Nitta, Takeshi Takahara, Hirokatsu Katagiri, Shoji Kanno, Akira Umemura, Yuji Akiyama, Takeshi Iwaya, Koki Otsuka, Akira Sasaki

**Affiliations:** ^1^ Department of Surgery Iwate Medical University School of Medicine Morioka City Japan

**Keywords:** hepatectomy, ischemic, laparoscopy, minimally invasive, steroid

## Abstract

**Aim:**

Laparoscopic liver resection (LLR) is increasingly carried out worldwide. However, there are concerns regarding ischemia‐reperfusion injury caused by pneumoperitoneum and the Pringle maneuver. It is not clear whether perioperative use of glucocorticoids lowers the risk of ischemia‐reperfusion hepatic injury in LLR as has been reported for open liver resection. The aim of the present study was to investigate the role of perioperative glucocorticoid use in improving hepatic function and surgical outcomes after LLR.

**Methods:**

In this double‐blind, randomized controlled trial (UMIN000013823), we enrolled 130 patients who presented to our institution for LLR between April 2014 and October 2018. Six patients were excluded, resulting in 124 patients being randomized to either the glucocorticoid or the control group. Preoperatively, patients in the glucocorticoid group received 500 mg methylprednisolone in saline solution, patients in the control group saline solution only. Surgical outcomes and blood parameters were compared between the two groups.

**Results:**

The Pringle maneuver could not be carried out in 24 patients, resulting in 50 patients in each group being included in the analysis. Postoperatively, total, direct and indirect bilirubin, and C‐reactive protein and interleukin‐6 levels were significantly lower, albumin levels were significantly higher, and prothrombin time was significantly shorter in the glucocorticoid than in the control group. Surgical outcomes were not significantly different between the groups.

**Conclusion:**

This first report on preoperative glucocorticoid use in LLR showed that it significantly improved postoperative liver function and thus might enhance the safety of LLR.

## Graphical abstract

In this single‐center, double‐blind, randomized controlled trial, we show the effect of a single bolus of methylprednisolone on hepatic function as evidenced by postoperative bilirubin and albumin levels, and prothrombin time after laparoscopic liver resection. We conclude that perioperative glucocorticoid use may enhance the safety of this procedure.

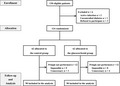

## 1 INTRODUCTION

Laparoscopic liver resection (LLR) is increasingly carried out worldwide because of recent technical advancements and clinical evidence of better short‐term and similar long‐term outcomes compared to open liver resection (OLR).^1^, ^2^, ^3^, ^4^, ^5^, ^6^ However, several studies have reported that pneumoperitoneum in laparoscopic surgery causes hepatic ischemia‐reperfusion injury as a result of the temporary decrease in portal venous blood flow.^7^, ^8^, ^9^ As LLR is a relatively new technique, reports on the prevention of hepatic injury during the procedure are scarce.

One of the major determinants of complications after hepatectomy is the extent of intraoperative bleeding. To reduce bleeding, the Pringle maneuver, which involves intermittent vascular clamping of the hepatic hilum, is commonly carried out. However, the resulting temporary hepatic ischemia and subsequent reperfusion leads to activation of complex metabolic, immunological, microvascular, and inflammatory processes that culminate in hepatocellular injury.^10^, ^11^, ^12^ This could be further aggravated by additional liver injury caused by pneumoperitoneum during LLR.

For OLR, several studies have shown that perioperative use of glucocorticoids may decrease the cytokine response to the ischemia‐reperfusion sequence and thus improve hepatic function and surgical outcomes.^13^, ^14^, ^15^, ^16^, ^17^ However, the effect of perioperative glucocorticoid use has not yet been evaluated for LLR. We hypothesized that giving perioperative glucocorticoid would improve postoperative hepatic function and morbidity in LLR. Therefore, our aim in the present study was to investigate the impact of glucocorticoid on hepatic function and surgical outcomes of LLR.

## 2 MATERIALS AND METHODS

### 2.1 Study design

This study was a single‐center, double‐blind, randomized controlled trial comparing preoperative glucocorticoid use to placebo in LLR carried out using the intermittent Pringle maneuver. This study was registered in the University Hospital Medical Information Network Clinical Trials Registry (UMIN000013823) and was approved by our institutional review board. Committee of Iwate Medical University School of Medicine, Approval No. H25‐179. The study conforms to the provisions of the Declaration of Helsinki. Informed consent was obtained from all subjects.

### 2.2 Participants

**Figure 1:**
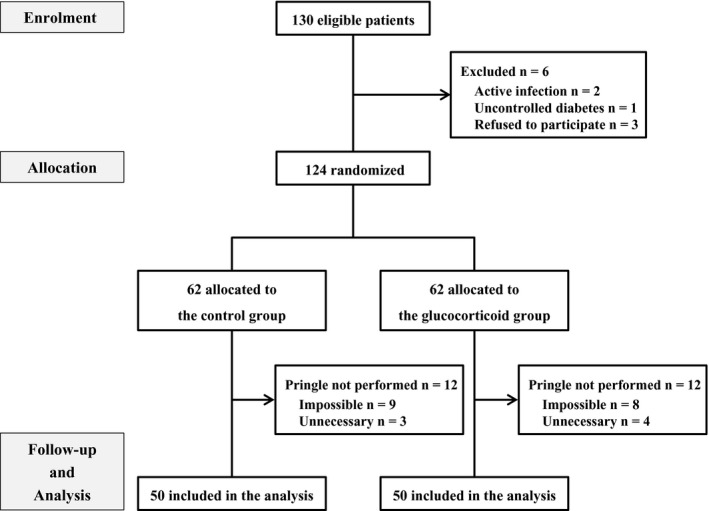
CONSORT flow diagram of the progress through the phases of the present randomized controlled trial. Patient enrolment (n = 130) and allocation between April 2014 and October 2018

Between April 2014 and October 2018, 130 patients who presented to our institution for LLR were enrolled into the trial. The CONSORT flow diagram is shown in Figure 1. Inclusion criteria were: (i) planned pure LLR using the intermittent Pringle maneuver; (ii) Eastern Cooperative Oncology Group performance status 0 or 1; (iii) age ≥20 years; (iv) no pregnancy. Exclusion criteria were: (i) active infection; (ii) uncontrolled diabetes mellitus; and (iii) refusal to participate.

Six patients were excluded before randomization based on these criteria (active infection [n = 2], uncontrolled diabetes mellitus [n = 1], and patient refusal to participate [n = 3]).

### 2.3 Randomization

The remaining 124 patients were randomly allocated to either the glucocorticoid (n = 62) or the control (n = 62) group using a 1:1 ratio. Group assignment was done by a statistician who was independent from our study using the minimization method program code in Microsoft Excel and Visual Basic for Applications (Microsoft Corporation). Patients who were allocated to the glucocorticoid group received 500 mg methylprednisolone dissolved in saline solution at the time of anesthesia induction. Patients in the control group received the saline solution only. Anesthesiologists were blinded to the study group to which patients belonged. Based on the random assignment by the statistician, another physician prescribed and prepared the infusion solution and handed it to the anesthesiologist who then gave it to the patient.

### 2.4 Baseline characteristics

Patient characteristics are reported in Table 1. No significant differences were identified between the two study groups.

**Table 1:** Baseline characteristics of 100 patients undergoing laparoscopic liver resection

	Control group (n = 50)	Glucocorticoid group (n = 50)	*P* value
Age (y)	68 (62‐75)	67 (59‐74)	.436
Male gender	31 (62.0)	30 (60.0)	>.999
Body mass index (kg/m^2^)	23.7 (21.1‐26.5)	24.1 (21.6‐26.6)	.540
Child‐Pugh Grade A	49 (98.0)	49 (98.0)	>.999
HBsAg positive	1 (2.0)	5 (10.0)	.204
HCV‐Ab positive	11 (22.0)	11 (22.0)	>.999
Liver cirrhosis	8 (16.0)	8 (16.0)	>.999
Total bilirubin (mg/dL)	0.5 (0.4‐0.7)	0.6 (0.4‐0.8)	.147
Direct bilirubin (mg/dL)	0.1 (0.1‐0.2)	0.2 (0.1‐0.2)	.389
Albumin (g/dL)	4.0 (3.9‐4.3)	4.1 (3.9‐4.3)	.124
AST (U/L)	26 (20‐37)	25 (20‐37)	.772
ALT (U/L)	24 (18‐36)	26 (16‐44)	.904
Prothrombin time (INR)	1.03 (0.98‐1.07)	1.02 (0.97‐1.07)	.580
Platelet count (×10^9^/L)	182 (133‐224)	183 (151‐242)	.620
C‐reactive protein (mg/dL)	0 (0‐0.2)	0 (0‐0.1)	.456
Interleukin‐6 (pg/dL)	2.5 (1.5‐5.1)	2.9 (1.6‐5.6)	.905
Fasting blood glucose (mg/dL)	102 (92‐113)	101 (95‐114)	.605
Hemoglobin A1c (%)	5.8 (5.5‐6.1)	5.7 (5.3‐6.0)	.225
Diagnosis (HCC/CRLM/Other)	26/14/10 (52.0/28.0/20.0)	23/21/6 (46.0/42.0/12.0)	.134
No. of tumors	1 (1‐1)	1 (1‐2)	.713
Size of tumor (mm)	34 (25‐43)	35 (24‐50)	.820

Glucocorticoid group, 500 mg methylprednisolone in saline preoperatively; control group, saline only.

Categorical variables are expressed as numbers (%) and continuous variables are presented as medians (interquartile range).

ALT, alanine aminotransferase; AST, aspartate aminotransferase; CRLM, colorectal liver metastasis; HBsAg, hepatitis B virus surface antigen; HCC, hepatocellular carcinoma; HCV‐Ab, hepatitis C virus antibody; INR, international normalized ratio.

### 2.5 Operative procedure

Patients were placed in the left half‐lateral decubitus position, or supine when the tumor was located in the left liver, and in a reverse Trendelenburg position. The anesthesiologist maintained low central vein pressure ≤3 cm H_2_O and low airway pressure ≤15 cm H_2_O. Carbon dioxide pneumoperitoneum was maintained at 10 mm Hg.

Liver parenchyma was transected using the clamp crush method, and a monopolar soft‐coagulation system was used for hemostasis. The intermittent Pringle maneuver was continuously repeated during parenchymal transection at a cycle of 15 minutes of clamping and 5 minutes of declamping. Patients in whom the Pringle maneuver could not be carried out because of either severe adhesions of the hepatoduodenal ligament or because of a change in operative method were consecutively excluded from the analysis.

### 2.6 Study endpoints

Primary endpoint was postoperative total serum bilirubin level. Secondary endpoints were serum aspartate aminotransferase (AST), alanine aminotransferase (ALT), C‐reactive protein (CRP), and interleukin‐6 (IL‐6) levels, prothrombin time, platelet count, and surgical outcomes. Blood analyses were done on the day prior to surgery and then daily on the first to the fifth postoperative day. Mid‐term follow‐up blood analyses were carried out once between the third and the fifth postoperative week.

### 2.7 Surgical outcome parameters

Extent of liver resection was classified according to The Brisbane 2000 Terminology of Liver Anatomy and Resections,^18^ and major hepatectomy was defined as a resection of three or more contiguous liver segments. Difficulty of LLR was evaluated using Hasegawa’s difficulty score.^19^ Postoperative morbidity and mortality were defined as any complication or death, respectively, occurring within 90 days of surgery. Complications were graded according to the Clavien‐Dindo classification system and were scored using the comprehensive complication index (CCI).^20^ The CCI was obtained using an online calculator (available at http://www.assessurgery.com). For example, a patient with one grade IIIa complication would have a CCI score of 26.2, whereas a patient with two grade II complications would have a CCI score of 29.6. Major morbidity was defined as CCI ≥26.2. Post‐hepatectomy liver failure (PHLF) was classified according to the definition proposed by the International Study Group of Liver Surgeries in 2011.^21^

### 2.8 Sample size

Based on our institutional data on LLR, we expected a standard deviation of the maximum total bilirubin level of 0.5 mg/dL. Sample size was calculated to detect a difference in total bilirubin level of 0.3 mg/dL,^15^ with type I error set at 0.05 (two‐sided), power at 0.80, and allocation ratio at 1:1. With these parameters, a sample size of 90 patients was required. Considering a rate of loss to follow up (including an impossible or unnecessary Pringle maneuver) of 30%, 130 patients were set as the target for enrolment.

### 2.9 Statistical analysis

Data were collected for all patients by one surgeon and confirmed by another surgeon to ensure the study protocol was followed. Continuous data are expressed as median values with the associated interquartile ranges. Categorical data are expressed as counts, with the associated percentile values calculated. The Wilcoxon rank‐sum test was used to compare continuous data, whereas Fisher’s exact test or the chi‐squared test was used for categorical data. *P* value <.05 was considered statistically significant. All statistical analyses were carried out using JMP statistical software version 9.0.0 (SAS Institute Inc.).

## 3 RESULTS

In the control group, 12 patients were excluded from analysis after surgery because the Pringle maneuver could either not be carried out (n = 9) or was not necessary (n = 3). Similarly, in the glucocorticoid group, 12 patients were excluded from the analysis because the Pringle maneuver could either not be carried out (n = 8) or was not necessary (n = 4). Ultimately, the analysis was based on the data of 50 patients each in the glucocorticoid group and in the control group.

Surgical outcomes are reported in Table 2. No significant differences were identified in these parameters between the two groups including the rate of PHLF (4.0% in both groups). However, the rate of morbidity and the CCI scores tended to be lower in the glucocorticoid group than in the control group. Median, 75th percentile, and 90th percentile levels of CCI were 0, 0, and 25.8 in the glucocorticoid group; and 0, 14.4, and 36.4 in the control group, respectively (*P* = .080).

**Table 2:** Surgical outcomes of 100 patients undergoing laparoscopic liver resection with intermittent Pringle maneuver

	Control group (n = 50)	Glucocorticoid group (n = 50)	*P* value
Major hepatectomy	11 (22.0)	10 (20.0)	>.999
Surgical difficulty (Low/Med/High)	14/25/11 (28.0/50.0/22.0)	11/27/12 (22.0/54.0/24.0)	.787
No. of hepatectomies during a surgery	1 (1‐1)	1 (1‐1)	.741
Operative time (min)	223 (157‐270)	215 (170‐294)	.677
Blood loss (mL)	34 (17‐76)	52 (29‐149)	.061
Transfusion	0 (0.0)	1 (2.0)	>.999
Time of Pringle maneuver (min)	60 (45‐84)	65 (49‐79)	.815
Conversion to open laparotomy	1 (2.0)	0 (0.0)	>.999
Hospital stay (days)	9 (7‐14)	9 (7‐13)	.615
Readmission	4 (8.0)	4 (8.0)	>.999
Morbidity	20 (40.0)	11 (22.0)	.083
Major morbidity	9 (18.0)	5 (10.0)	.388
Mortality	0 (0.0)	0 (0.0)	>.999
CCI score	0 (0‐14.4)	0 (0‐0)	.080
PHLF grade ≥ B	2 (4.0)	2 (4.0)	>.999

Glucocorticoid group, 500 mg methylprednisolone in saline preoperatively; control group, saline only.

Categorical variables are expressed as numbers (%) and continuous variables are presented as medians (interquartile range).

CCI, comprehensive complication index; PHLF, post‐hepatectomy liver failure.

**Figure 2:**
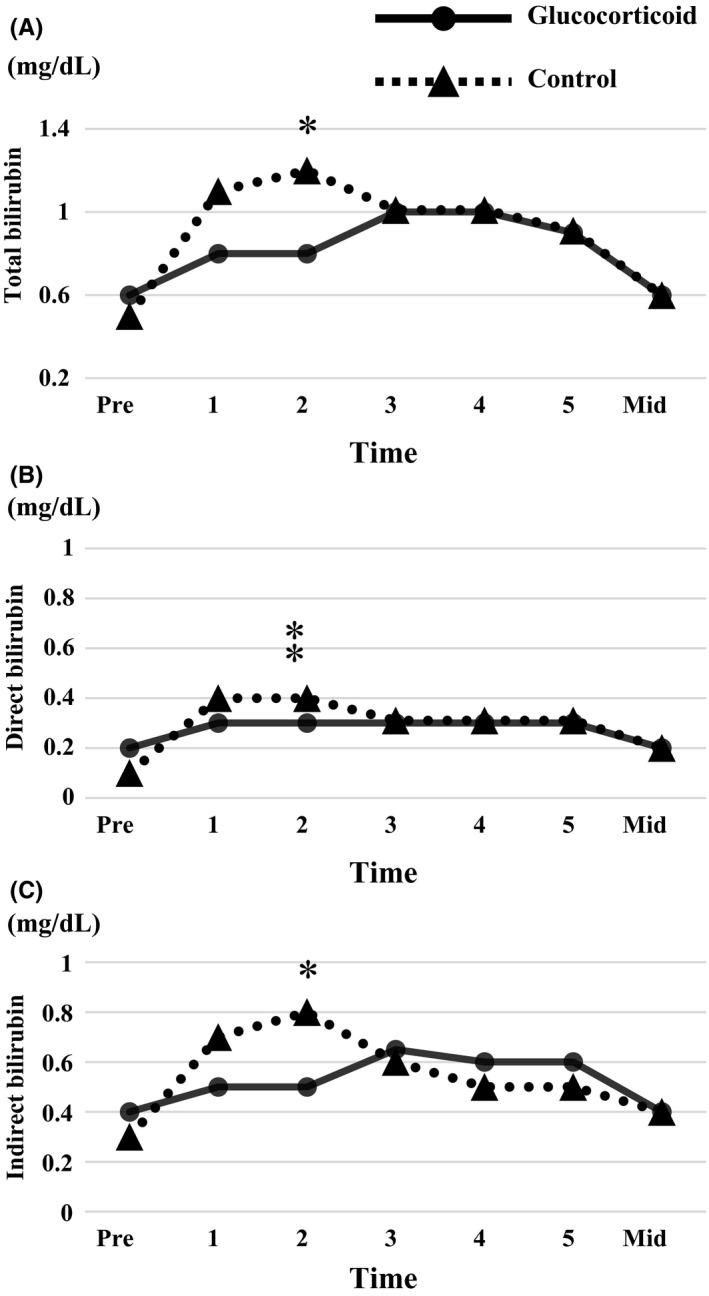
Pre‐ and postoperative bilirubin levels in 100 patients undergoing laparoscopic liver resection with intermittent Pringle maneuver. A, Total bilirubin, B, Direct bilirubin, C, Indirect bilirubin. *P < .01, **⁑** *P* < .001. Pre, preoperatively; 1,2,3,4,5, days postoperatively; Mid, third to fifth week postoperatively

**Figure 3:**
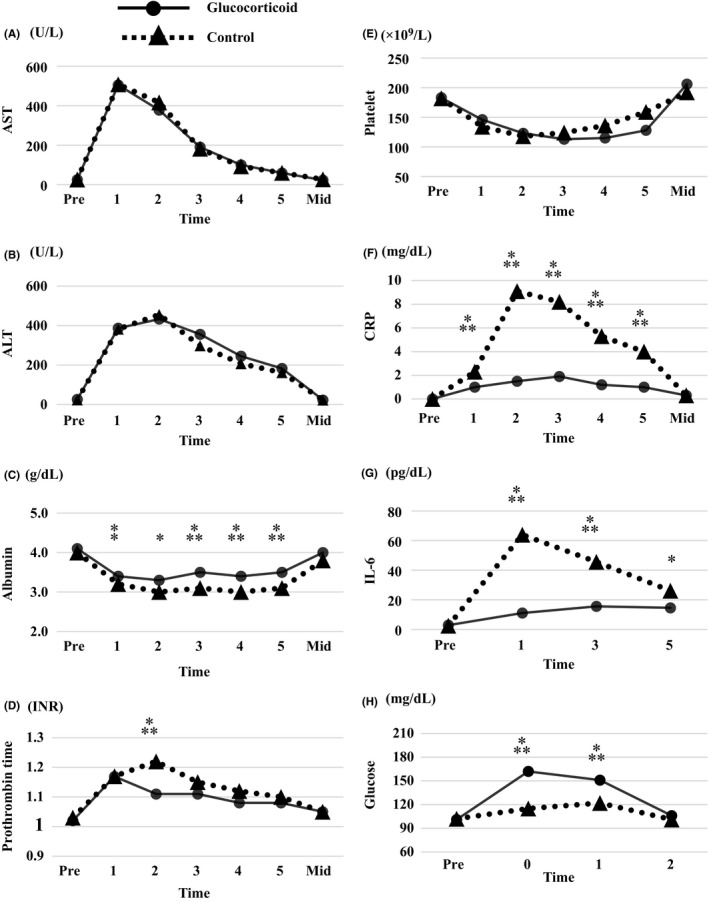
Pre‐ and postoperative blood parameter levels in 100 patients undergoing laparoscopic liver resection with intermittent Pringle maneuver. A, aspartate aminotransferase; B, alanine aminotransferase; C, albumin; D, prothrombin time; E, platelet count; F, C‐reactive protein; G, interleukin‐6, H, fasting blood glucose. *P < .01, ⁑*P* < .001, ⁂*P* < .0001. Pre, preoperatively; 1,2,3,4,5, days postoperatively; Mid, third to fifth week postoperatively

Time course of total, direct, and indirect bilirubin levels before and after surgery is shown in Figure 2. Total, direct, and indirect bilirubin levels on the second postoperative day were significantly lower in the glucocorticoid than in the control group. Time course of other blood analyses is shown in Figure 3. The lowest level of albumin (3.2 g/dL vs 2.9 g/dL, *P* = .0002) was significantly higher, the longest prothrombin time expressed as the international normalized ratio (INR) (1.19 vs 1.23, *P* = .035), and the highest levels of CRP (2.1 mg/dL vs 9.1 mg/dL, *P* < .0001) and IL‐6 (31.2 pg/dL vs 80.9 pg/dL, *P* < .0001) were significantly lower in the glucocorticoid than in the control group, respectively. Peak IL‐6 values were reached on postoperative day 1 in 58% of patients in the control and 24% in the glucocorticoid group, on day 3 in 24% of patients in the control and 48% in the glucocorticoid group, and on day 5 in 18% of patients in the control and 28% in the glucocorticoid group. Levels of fasting blood glucose were higher among patients in the glucocorticoid than in the control group until the first postoperative day, recovering to similar levels as in the control group on the second postoperative day.

## 4 DISCUSSION

This is the first randomized controlled trial to report the impact of giving preoperative glucocorticoid on liver function and surgical outcomes after LLR. Previous studies reported that glucocorticoid use in OLR had a positive effect on postoperative complications and blood parameters.^13^, ^14^, ^15^, ^16^, ^17^ In a meta‐analysis of glucocorticoid use for OLR, methylprednisolone was used in five studies and hydrocortisone in one study.^14^ All six studies showed positive impacts of giving glucocorticoid. We chose methylprednisolone for our study because it has less mineralocorticoid activity and a longer half‐life than hydrocortisone. Our study showed that a single preoperative i.v. bolus of methylprednisolone in LLR significantly lowered postoperative total bilirubin level, increased albumin, reduced CRP and IL‐6 levels, and shortened prothrombin time, confirming our hypothesis that it would improve postoperative liver function. There was also a trend toward better surgical outcomes.

Post‐hepatectomy liver failure is one of the most serious complications of liver surgery, with postoperative total bilirubin levels and prothrombin time being indicative of PHLF.^21^, ^22^ In the present study, the rate of PHLF was generally low and comparable between the glucocorticoid and control groups. However, we believe that the improvement in postoperative total bilirubin level and prothrombin time owing to glucocorticoid use may have a positive clinical impact on patient outcome.

The intermittent Pringle maneuver, a temporary clamping of the hepatoduodenal ligament, is a safe and useful procedure to reduce blood loss during liver resection. However, the intermittent Pringle maneuver does carry a risk of ischemia‐reperfusion injury to the liver, which is defined as an inflammatory response and organ damage, triggered by ischemia inducing hypoxic stress and reperfusion inducing oxidative stress.^23^, ^24^ Numerous factors contribute to hepatic ischemia‐reperfusion injury, with activation of Kupffer cells, induction of oxidative stress, and upregulation of proinflammatory cytokine signaling.^25^, ^26^ Raised blood levels of cytokines are associated with an increased incidence of postoperative infection and organ dysfunction.^26^, ^27^ IL‐6 is considered an integral mediator of the acute‐phase response, and CRP is considered an index of the inflammatory response. We showed that giving a bolus of methylprednisolone prior to the start of LLR suppressed the inflammatory response, with postoperative levels of IL‐6 and CRP being significantly lower in the glucocorticoid than in the control group. Of note, the suppression of cytokinemia might decrease vascular permeability,^28^ which could explain the higher postoperative levels of albumin in the glucocorticoid group.

Association between laparoscopic surgery and a postoperative decrease in liver function has previously been reported.^9^, ^29^ This decrease in liver function likely results from pneumoperitoneum pressure, which is usually higher than the normal portal blood pressure, leading to a reduction in portal blood flow.^7^, ^30^ The inflation and deflation of the pneumoperitoneum may also induce an ischemia‐reperfusion injury. Thus, there are two possible causes of hepatic ischemia‐reperfusion injury during LLR, one being the intermittent Pringle maneuver and the other the pneumoperitoneum. Therefore, greater care is required to prevent ischemia‐reperfusion hepatic injury in LLR than in OLR. Additionally, the operative time is often longer for LLR than for OLR,^5^, ^6^ resulting in a longer overall duration of the Pringle maneuver. Thus, giving glucocorticoid might have a greater effect in LLR than in OLR.

Regarding the postoperative complications, we had hypothesized that glucocorticoid use would decrease postoperative morbidity through its attenuation of the inflammatory response after major abdominal surgery, which is a main contributor to postoperative morbidity and delays recovery.^31^, ^32^ The absence of a significant difference in postoperative morbidity between the glucocorticoid and control groups in our study might be related to the relatively small number of patients (n = 50) in each group. However, we did observe a trend towards lower morbidity rates and CCI scores in the glucocorticoid as compared to the control group. Lower levels of postoperative CRP and IL‐6 may further contribute to decreasing the incidence of postoperative complications.

However, increase in blood glucose levels is an important adverse effect of glucocorticoid use that must be considered. On the first postoperative day, among patients in the glucocorticoid group, blood glucose level was higher than normal, and higher than the blood glucose values in the control group, returning to normal levels by the second postoperative day. Therefore, in the present study, the adverse effect of the methylprednisolone bolus on blood glucose levels was considered to be transient and limited.

Limitations of the present study must be considered in the interpretation of the results. This was a single‐center trial with a relatively small number of patients and, therefore, selection bias cannot be excluded. Because of the small sample size, subgroup analyses to evaluate potential effects of the extent of hepatectomy, liver cirrhosis and the length of the Pringle maneuver on postoperative blood parameters could not be carried out. Therefore, larger scale, multicenter studies are required to confirm our findings.

In conclusion, this is the first report on the effect of giving a single bolus of preoperative methylprednisolone on hepatic function and surgical outcomes after LLR, showing a benefit when assessing postoperative bilirubin and albumin levels, and prothrombin time. Based on our results, we believe that preoperative glucocorticoid use could enhance the safety of LLR, which is increasingly carried out worldwide.

## DISCLOSURE

Funding: There were no funding supports for this study.

Conflicts of Interest: Authors declare no conflicts of interest for this article.

Author Contribution: All authors: conception, design, interpretation of data, surgery, and acquisition of data. Y.H. and T.T.: data analysis. 
